# Change of Body Composition and Adipokines and Their Relationship with Insulin Resistance across Pubertal Development in Obese and Nonobese Chinese Children: The BCAMS Study

**DOI:** 10.1155/2012/389108

**Published:** 2012-12-11

**Authors:** Lu Xu, Ming Li, Jinhua Yin, Hong Cheng, Miao Yu, Xiaoyuan Zhao, Xinhua Xiao, Jie Mi

**Affiliations:** ^1^Endocrine Key Laboratory of Ministry of Health, Department of Endocrinology, Peking Union Medical College Hospital, Peking Union Medical College and Chinese Academy of Medical Sciences, No. 1 Shuaifuyuan, Wangfujing, Beijing 100730, China; ^2^Department of Epidemiology, Capital Institute of Pediatrics, Beijing 100020, China

## Abstract

A transient increase in insulin resistance (IR) is a component of puberty. We investigated the impact of body composition and adipokines on IR during puberty in Chinese children. This study included 3223 schoolchildren aged 6–18 years. IR was calculated using homeostasis model assessment (HOMA-IR). We revealed that body mass index (BMI) and waist circumference increased gradually during puberty in both genders, while fat-mass percentage (FAT%) increased steadily only in girls. Change of leptin showed striking sexual dimorphisms: in girls leptin increased steadily during puberty, whereas in boys, after a transient rise at the beginning of puberty, leptin declined by Tanner staging even in those overweight or obese. Inversely, adiponectin level decreased significantly during puberty. In both genders, HOMA-IR started to increase at the beginning of puberty, peaked in the middle, and revised at late puberty in overweight/obesity boys while it stayed high till the end of puberty in girls and normal weight boys. Multivariate regression analysis revealed that leptin presented a stronger indicator of HOMA-IR than anthropometric measures during puberty. Our results demonstrated that gender-specific FAT% and leptin changed with pubertal development. Leptin emerged as a stronger predictor of IR than traditional anthropometric indices, suggesting a prominent role in the development of pubertal IR.

## 1. Introduction

Childhood obesity has become a global epidemic problem [1]. Obesity related morbidities during pubertal transition have aroused the attention of more physicians. Recently, a growing body of evidence has suggested that a transient increase in insulin resistance is a component of pubertal development. There is a phenomenon of increasing insulin resistance that begins in early puberty, peaks in the mid of puberty, and resolves by the end of puberty in normal weight children [2–5]. However, data about the insulin resistance pattern during adolescence in obese subjects has been discordant [6, 7]. Given that insulin resistance is a feature of obesity and hallmarks metabolic syndrome, moreover, puberty is a high risk developmental period for obesity related disease, it is important to understand the modulators of pubertal insulin resistance.

It has been well accepted that adipose tissue is a dynamic endocrine organ releasing a number of adipocyte-specific factors named “adipokines," which are critical for regulating metabolism in both health and disease. Of various adipokines, leptin and adiponectin have been proposed to be a link between adipose tissue and insulin resistance. Leptin expression and plasma concentrations have been shown to be proportional to adipose tissue mass in humans and are therefore increased in adiposity [8, 9] and reduced after weight reduction in both adults [10, 11] and children [12, 13]. With the anti-inflammatory, antiapoptotic, and proangiogenic properties, adiponectin is one of the most potent adipokines with respect to its insulin-sensitizing activity [14, 15].

In the light of growing obesity epidemics in children and adolescents worldwide, further studies on adipokines and insulin resistance during puberty are clearly warranted. To our knowledge, despite well-observed gender differences in body composition and adipokines during adolescents [3–5, 16], few studies have examined gender-specific association of adipokines alternations with puberty insulin resistance.

Therefore, the aim of our study was to assess the correlation of the dynamics of insulin resistance with leptin and adiponectin, as well as anthropometric measures of body fat during puberty in a large sample of Chinese children and adolescents.

## 2. Materials and Methods

### 2.1. Subjects

Subjects were recruited from a cross-sectional population-based survey: the Beijing Child and Adolescent Metabolic Syndrome (BCAMS) study as described elsewhere [17]. The cohort contained 3530 children aged 6–18 years and classified as normal weight (BMI ≤ 85%), overweight (BMI > 85% but ≤95%), and obese groups (BMI ≥ 95%) according to age- and gender-specific BMI percentiles cutoffs defined by the Working Group on Obesity in China [18]. However, a total of 3223 children including 1616 boys and 1607 girls who met the following eligibility criteria were included for the current analysis: (1) subjects without missing data; (2) lack of diabetes mellitus and other underlying chronic diseases; (3) no current regular medications. Informed consents from participants and/or parents/guardians were obtained before entering into the study. The BCAMS study was approved by the Ethics Committee at Capital Institute of Pediatrics in Beijing.

### 2.2. Anthropometric Parameters and Biochemical Analyses

A detailed description of the sample collection and detection procedures has been previously published [17]. In short, subjects were evaluated for body mass index (BMI), BMI z-score by age and gender, waist circumference, fat-mass percentage and (FAT%), systolic and diastolic blood pressure (SBP and DBP). BMI and FAT% were used as measures of general adiposity, whereas waist circumference was used as measures of central adiposity. Waist circumference was measured midway between the lowest rib and the superior border of the iliac crest with an inelastic measuring tape at the end of normal expiration to the nearest 0.1 cm. FAT% was assessed by bioelectrical impedance analysis (BIA, TANITA TBF-300A). Venous blood samples were collected by direct venipuncture after an overnight (minimum 12 h) fast. The samples were centrifuged, aliquoted, and immediately frozen and stored at −80°C for future analysis of lipids and hormones. Serum lipids including triglyceride (TG), total cholesterol (TC), high-density lipoprotein cholesterol (HDL-C), low-density lipoprotein cholesterol (LDL-C) (enzymatic methods), and plasma glucose (glucose oxidize method) were assayed using the Hitachi 7060 C automatic biochemistry analysis system. HDL-C and LDL-C were measured directly. Serum insulin, adiponectin, and leptin were measured by sandwich enzyme-linked immunosorbent assays (ELISA) [19], which were developed and performed centrally in Key Laboratory of Endocrinology in Peking Union Medical College Hospital. The insulin assay had a sensitivity of 0.5 mU/L and an interassay CV of <9.0%, and no cross-reactivity to proinsulin (<0.05%). Assay characteristics for the measurement of adiponectin and leptin were as follows: intra-assay CVs of <5.4% and <7.4%, respectively; interassay CVs of <8.5% and <9.3%, respectively [17, 20].

### 2.3. Pubertal Stage

Pubertal development was assessed by Tanner stage of breast development (girls) and testicular volume (boys) [21]. This assessment was performed visually by two pediatricians of the same gender as the child. Subjects were divided into gender and Tanner stage (T) as follows: prepubertal (T1), early pubertal (T2), midpubertal (T3-4), and late pubertal (T5).

### 2.4. Calculation and Statistical Analysis

BMI was calculated as weight (kg) divided by height squared (m^2^). Insulin resistance index was calculated by homeostasis model assessment of insulin resistance (HOMA-IR) as follows: fasting insulin (mU/L) × fasting glucose (mmol/L)/22.5 [22]. Unless otherwise stated, all results were displayed as mean ± standard error (SE). All data analyses were performed using SPSS15.0 (SPSS Inc., Chicago, IL, USA). Statistical normality of the data distribution was verified using the Kolmogorov-Smirnov test. Nonparametric data was analyzed after being logarithmically transformed. Student's *t*-test was used for comparison of two groups. One-way ANOVA was used for comparison of multiple groups (as indicated) with *post hoc* comparison being used to evaluate group differences. For contribution of multiple independent variables on one dependent variable, multiple regressions using forward stepwise regression analysis were used. A *P* value of 0.05 was considered statistically significant for all analysis.

## 3. Results

### 3.1. Anthropometric and Metabolic Characteristics

Clinical and metabolic data of the subjects separated according to sex and weight are shown in Table 1. Between boys and girls, in normal weight group, differences existed in most parameters except diastolic blood pressure (DBP), HDL-C, and adiponectin. In both overweight and obesity groups, boys and girls had similar age, diastolic blood pressure, and levels of adiponectin and lipids (TC, TG, HDL-C, LDL-C). Moreover, there was no BMI difference between overweight boys and girls, whereas obesity boys had larger BMI value than obesity girls. Boys had greater BMI z-score than girls in each weight group. Within either sex, BMI, BMI z-score, waist circumference, FAT%, SBP, DBP, and levels of TG, leptin and HOMA-IR values increased with adiposity, while HDL-C and adiponectin decreased with adiposity.

### 3.2. Body Composition and Pubertal Development

**Figure 1:**
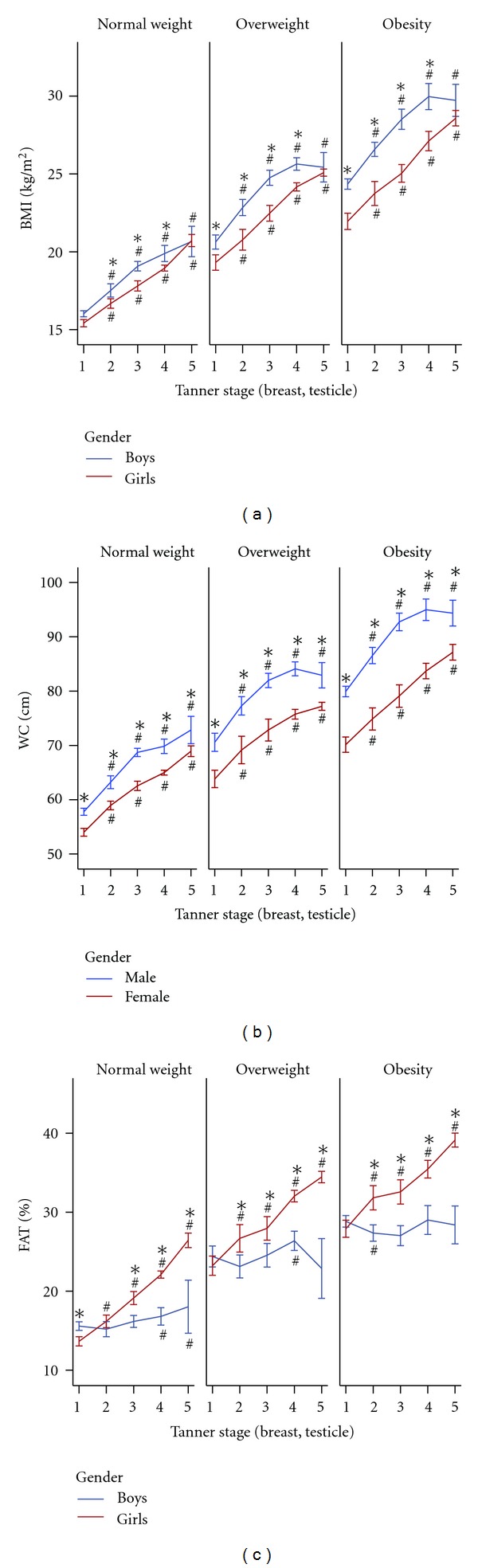
(a) Body mass index (BMI), (b) waist circumference (WC), (c) fat mass percentage (FAT%) and pubertal development. Data were expressed as means ± 95% CI. ^#^ *P* < 0.05 compared with T1 of the same gender; *P < 0.05 compared between boys and girls of the same Tanner stage. Two-sample *t*-test for comparison between girls and boys and one-way ANOVA with five groups (T1 to T5) to compare characteristics across these five Tanner stages, were used.

Children and adolescents were separated according to BMI (normal weight, over weight, and obesity), Tanner stage, and gender (Figure 1(a)). In all three weight groups (normal weight, over weight, and obesity), BMI increased throughout puberty in both genders, with faster increment occurring in early puberty (T1–T3). From T1 to T4, boys had higher BMI than girls in all three groups (*P* < 0.001; Figure 1(a)), but at the end of puberty (T5), the difference in BMI disappeared between boys and girls. Measurement of waist circumference rose steadily across pubertal stage in all groups except a slight falling from T4 to T5 in overweight and obesity boys (Figure 1(b)). However, unlike BMI, waist circumference was significantly higher in boys than in girls at each T stages regardless of weight. As shown in Figure 1(c), in all three groups, FAT% increased continuously with puberty progression in girls but exhibited almost no change in boys, thus reached the biggest difference at the end of puberty (T5) between the two genders.

### 3.3. Leptin, Adiponectin, and Pubertal Development

**Figure 2:**
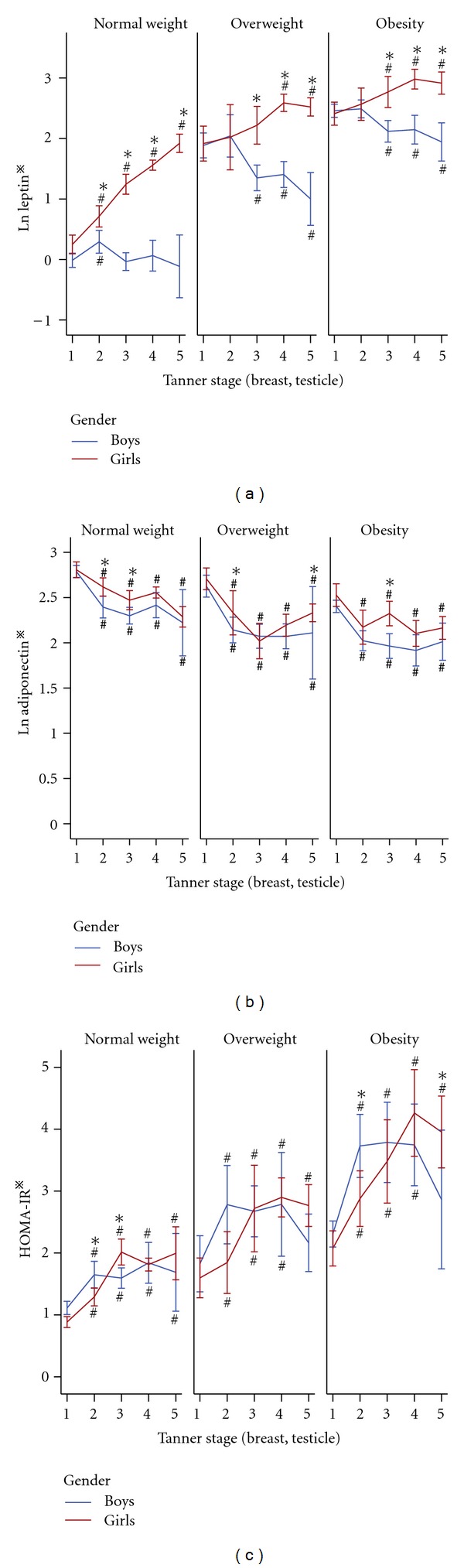
(a) Leptin, (b) adiponectin, (c) insulin resistance (HOMA-IR) and pubertal development. Data are expressed as means ± 95% CI. ^#^ *P* < 0.05 compared with T1 of the same gender; *P < 0.05 compared between boys and girls of the same Tanner stage. Statistical methods as in Figure 1. *Skewed distributions were logarithmically transformed for comparison.

Figure 2(a) showed that in all three weight groups, leptin levels in girls increased steadily with pubertal development. Its levels in boys raised parallel to that in girls in early puberty, then, after peaking at T2, it decreased till the end of puberty especially in those overweight or obese. Differences of leptin levels between two genders were significant at T2–5 (*P* < 0.001) in normal weight group, at T3–5 (*P* < 0.001) in overweight and obesity groups. Before puberty, serum adiponectin levels had no significantly difference between girls and boys in all three groups (Figure 2(b)). The levels, then, declined with pubertal procession for both sexes, with faster decline rate in boys. Consequently, adiponectin levels were lower in boys than that in girls during puberty, and the differences were significant at T2 (*P* < 0.01) and T3 (*P* < 0.05) in normal weight, T2 (*P* < 0.05) and T5 (*P* < 0.05) in overweight, and T3 (*P* < 0.01) in obesity group, respectively.

### 3.4. HOMA-IR and Pubertal Development

As shown in Figure 2(c), HOMA-IR indices of boys and girls increased with the onset of puberty, reaching the highest value at T3-T4 (*P* < 0.001), almost twice of that at T1 in all three weight groups. However, the HOMA-IR indices of boys and girls decreased at T4-5 in overweight and obesity groups, while stayed high till the end of puberty in normal weight group. At the end of puberty (T5), indices of HOMA-IR in overweight and obesity boys were similar to that before puberty. In spite of the falling from T4 to T5, indices of HOMA-IR at T5 were still higher than that before puberty in overweight and obesity girls (*P* < 0.01). As a whole group, girls had lower HOMA-IR indices than boys in early puberty, but not in mid- and later puberty. Obviously, HOMA-IR values of both genders in obesity and overweight groups were higher than that in normal weight group at any T stage (all *P* < 0.01).

### 3.5. Multiple Linear Regression Analysis for Dependent Variable HOMA-IR

Firstly, multivariate analyses for HOMA-IR were stratified by sex and BMI (Table 2). Leptin showed a strong and independent predictor of HOMA-IR, in boys (*P* < 0.001) and girls (*P* < 0.001), regardless of obesity status. Adiponectin was an independent determinant of HOMA-IR in overweight (*P* = 0.023) and obesity boys (*P* = 0.001) and overweight girls (*P* = 0.01). Waist circumference was independently associated with HOMA-IR in obesity children (boys, *P* = 0.006, girls, *P* < 0.001). Replacing waist circumference with FAT% or BMI z-score did not affect the contribution significance of adipokines to HOMA-IR. However, FAT% was a contributor to HOMA-IR only for obese girls (beta: 0.126, *P* = 0.034) and BMI z-score only for obese children (beta: boys, 0.086, *P* = 0.027; girls, 0.140, *P* = 0.003) (data not shown). Moreover, Tanner staging showed independent contributor to HOMA-IR in all groups except in obese girls.

Secondly, to identify which parameters were better indices to predict insulin resistance during puberty, we further separated the subjects by sex and pubertal development (Table 3). In boys, leptin and waist circumference contributed more or less the same to HOMA-IR (beta value: 0.253 versus 0.207) before puberty. After the onset of puberty, leptin became a prominent determinant of HOMA-IR (beta at T2: 0.407, T3: 0.515, *P* < 0.001, resp.), and adiponectin (*P* = 0.001) emerged as a contributor to HOMA-IR in midpuberty. In late puberty, beta value of leptin decreased, and waist circumference and adiponectin were no longer determinants of HOMA-IR. In girls, leptin represented a stronger contributor to HOMA-IR than waist circumference, while adiponectin was not an independent contributor to HOMA-IR throughout puberty. When FAT% or BMI z-score was inserted as a covariate instead of waist circumference, the relationship between leptin and HOMA-IR did not change (data not shown). In boys, FAT% was not a predictor for HOMA-IR at any time. However, in girls, FAT% was independently associated with HOMA-IR before and in late puberty. BMI z-score showed independent association with HOMA-IR only before puberty in boys.

## 4. Discussion

To our knowledge, the present cross-sectional analysis is the largest study on the relationship of the pubertal insulin sensitivity with Tanner stage, sex, body composition, and adipokines in nonobese and obese children and adolescents to date. Out data demonstrated that in both nonobese and obese children, HOMA-IR index increased significantly in mid-Tanner stage, confirming the presence of insulin resistance in puberty [2–7]. Moreover, BMI, waist circumference, FAT%, and levels of leptin and adiponectin in serum changed with the progression of puberty, and those changes were of sex differences.

In the current study, although BMI and waist circumference in both genders increased progressively with pubertal development, body composition modifications were sexually dimorphic: FAT% increased with puberty in girls but not in boys. This sexual difference suggests that the accumulation of fat in girls may largely explain the accretion of BMI during puberty but in boys the increment in BMI may be mainly attributed to accumulation of fat-free tissue. Moreover, gender differences in BMI disappeared at the end of puberty, while differences in waist circumference remained at late puberty, with boys having higher waist circumference than girls. Since it has been reported that in children waist circumference is more associated with visceral fat [23], our results indicate that boys tend to have a more fat distributed prominently in the abdominal area during the pubertal process. Gender itself also influences the change pattern of leptin levels: in our study, leptin levels in girls increased steadily with progression of puberty; however, in boys, the levels of leptin increased transiently at the beginning of puberty, followed by a decrease till late puberty particularly in those overweight or obese. Consequently, girls had on average twice as much leptin as boys at the end of puberty. It has been known that leptin circulates at levels directly proportional to body fat; however, in boys, FAT% measured in our study did not decrease like leptin, but was stable during puberty even in those overweight or obesity. This dissociation might be explained by the method measuring fat mass which is not able to differentiate subcutaneous fat tissue and visceral fat tissue. Bulk of evidence have showed that leptin mRNA expression is enhanced in subcutaneous located adipocytes compared to visceral adipocytes [24, 25] and that circulating levels of leptin correlate with subcutaneous and total (not visceral) adipose tissue [26]. This is in consistence with our founding in girls that change of BMI, FAT%, and leptin was similar, because adipose tissue in girls might develop prominently in the subcutaneous depots. Inversely, in boys, body fat percent measured by more accurate (method i.e., dual energy X-ray absorptiometry), has been found to be decreased during puberty [27]. Furthermore, as indicated by waist circumference in our study, males, relative to females, tend to partition fat to the visceral spaces [28]; they would be expected to have lower serum leptin concentrations than females. Indeed, our study certificated that leptin levels declined in overweight and obesity boys in puberty. Thus, gender differences in both body composition and body fat distribution during puberty lead to the variations of serum leptin concentrations. However, due to the limitations of the BIA method, we could not investigate the relationship between leptin levels and different fat depots in our subjects. Moreover, gender differences in synthesis, transport, or clearance of the leptin [29] and a potential role of gonadal steroids also contribute to the gender distinction of serum leptin [30]. It has been reported that circulating concentrations of testosterone significantly negatively correlate with circulating concentrations of leptin normalized to fat mass in males but not in females [30]. Studies in both human and rodents have suggested that the inhibition by circulating androgens is one reason to explain the lower levels of leptin in males [31]. In addition, the summit of leptin level at the onset of puberty in boys can be interpreted by the permissive action of leptin on trigger of puberty [32].

In our analysis, in both sexes, there was a significant decrease in adiponectin levels as children transitioned to puberty. Boys had lower levels of adiponectin than girls in puberty. Our data were consistent with previous studies which have reported that at the beginning of puberty, adiponectin levels are similar in boys and girls. However, most studies show that adiponectin levels in healthy girls remain almost no change during the pubertal process [33–36], although Woo et al. have reported a fall in obese girls [36]. On the other hand, in boys, similar to our findings, it has been revealed that adiponectin levels decrease in midpuberty (between T2 and T3) and remain low till the end of puberty. From midpuberty onwards, adiponectin levels in boys are significantly lower than those in girls. [33–36]. The falling of adiponectin levels in boys have been most likely due to rising androgen levels. According to two studies of pubertal children [33, 37], adiponectin levels are inversely correlated with androgen concentrations (and particularly testosterone in boys). Higher testosterone production at increased Tanner staging leads to a decrease of sex hormone binding globulin which is positively associated with adiponectin [35], and moreover, administration of testosterone to hypogonadal males has been shown to suppress adiponectin levels [38]. Otherwise, similar to leptin, human adiponectin gene expression has been found to be lower in visceral compared to subcutaneous fat [39], and this might be another reason for the decline of adiponectin level in boys. The decrease of adiponectin in girls observed in our study possibly resulted from the increase in fat mass percent in this period. Briefly, gender difference in adiponectin level during puberty is strongly associated with serum androgen concentrations and sexual dimorphism of changes in body composition and fat distribution.

Although pubertal insulin resistance has been observed and studied for many years, the cause of it is uncertain [16, 40]. In the present study, we developed multivariate regression analysis to determine which parameter, either adiposity, body composition, or adipokines, was more related to the maturational increases in HOMA-IR. In the analysis, we stratified our subject by gender, weight, and puberty to specify the associations of the above parameters with HOMA-IR. We found that leptin was an independent determinant of the pubertal insulin resistance. And, regardless of sex and obesity status, leptin emerged as a stronger contributor to pubertal HOMA-IR than FAT%, waist circumference, or BMI z-score, which were traditional measurements of obesity. However, it is intriguing when looking at the trends of leptin concentration and HOMA-IR, which were very different during puberty. Thus it should be noted that although leptin in our study appeared more related to HOMA-IR, its contribution to puberty insulin resistance might still be limited, because leptin together with all these parameters observed in multivariate analysis could only explain less than half of the total variance of HOMA-IR (i.e., *R* ^2^ ranged at 0.33–0.48).

Associations between adiponectin and insulin resistance have been controversial between studies. Some studies have reported moderately strong inverse correlations, independently of potential confounders including age, race, sex, total fat, and Tanner stage [41], while others have not shown associations after being adjusted for body weight [38]. In our study, when were the children grouped by sex and puberty, the relationship between falling adiponectin level and HOMA-IR varied with sex, pubertal development, and other covariates in the models (different anthropometry measurements: BMI, waist circumference or FAT %). In the weight-stratified groups, adiponectin presented as an independent indicator of HOMA-IR in overweight and obesity boys and overweight girls but not in lean subjects. That particularly central obesity, exerted an effect on the relationship of adiponectin with insulin resistance.

Moreover, among traditional adiposity indicators, waist circumference was more related to HOMA-IR than BMI z-score and FAT% during puberty in both genders. In agreement with our results, waist circumference has been found to be an independent predictor of insulin resistance in black and white youth [42]. It has been stated that in children, waist circumference is more associated with visceral fat, whereas BMI is more related to subcutaneous fat [23]. Interestingly, only visceral fat is associated with fasting insulin and TG in obese adolescent girls [43]. Thus, waist circumference, a marker of abdominal obesity, has been recommended as an important component of pediatric metabolic syndrome definition [44]. However, it also should be noted that the limitations of the BIA method employed by our study to measure FAT% might be an important reason of bias in considering the contribution of boy composition to HOMA-IR. Nonetheless, although the traditional simple measurements of adiposity could not be substituted for their contribution to pubertal insulin resistance, the adipose-derived adipokines, especially leptin, rather than those traditional adiposity indicators, like BMI, waist circumference, or FAT%, are more sensitive to detect high-risk individuals of metabolic syndrome at an early age. Therefore, monitoring these adipokines as a supplement to anthropometric measurement would be expected to provide important information for assessing metabolic risks as early as in childhood.

The major strength of this study was that we comprehensively evaluated the relative role of anthropometric measurements, biochemical parameters, and adipokines in predicting insulin resistance estimated by HOMA-IR in puberty in a large representative sample of Chinese children. Nevertheless, several limitations of our study should be acknowledged. First, the BIA method we used cannot distinguish visceral from subcutaneous fat and barely assessed the effect of different adipose deposit on pubertal insulin resistance. Second, the results were limited by the use of HOMA rather than the gold standard technique, that is, hyperinsulinemic-euglycemic clamp, for its complexity in large pediatric population. Third, cause-and-effect relationships could not be inferred because of the cross-sectional nature of the study design. Despite these limitations, our investigation provides some robust cross-sectional data on metabolic and anthropometric characteristics and their association with pubertal insulin resistance of obese and nonobese Chinese individuals during pubertal development.

## 5. Conclusions

In conclusion, both obese and nonobese children experience a stage of decreased insulin sensitivity during puberty. BMI, waist circumference, FAT%, and adipokines change with the progression of puberty, and these changes are of sex dimorphism. Increase of leptin level is a reliable indicator of the pubertal insulin resistance as estimated by HOMA-IR, and adiponectin can be envisioned as obesity-associated predictor of insulin resistance in puberty. Therefore, in pubertal children, increase in serum leptin level and decrease in adiponectin level may allow the early identification of “at-risk” individuals, providing important prognostic information in predicting insulin resistance and metabolic syndrome. However, longitudinal studies are necessary to evaluate the role of adipokines in the normal process of growth and in the development of obesity-related disease in childhood.

## Figures and Tables

**Table 1:** Anthropometric and metabolic characteristics of the children and adolescents according to sex and weight status.

	Boys			Girls		
	Normal weight	Over weight	Obesity	Normal weight	Over weight	Obesity
*n*	608	305	703	892	308	407
Age (years)	11.7 ± 0.1	12.7 ± 0.2	11.3 ± 0.1	12.4 ± 0.1^c^	12.9 ± 0.2	11.3 ± 0.1
BMI (kg/m^2^)	17.7 ± 0.1	23.3 ± 0.2^a^	26.9 ± 0.1^ab^	18.1 ± 0.1^c^	23.5 ± 0.1^a^	25.9 ± 0.2^abc^
BMI-z*	−1.02 ± 0.02	0.04 ± 0.02^a^	0.90 ± 0.02^ab^	−0.7 ± 0.02^c^	0.5 ± 0.02^ac^	1.2 ± 0.04^abc^
FAT%	16.6 ± 0.2	24.6 ± 0.3^a^	28.8 ± 0.2^ab^	19.9 ± 0.2^c^	31.0 ± 0.3^ac^	34.4 ± 0.3^abc^
Waist circumference (cm)	63.3 ± 0.3	78.3 ± 0.5^a^	85.8 ± 0.4^ab^	62.2 ± 0.2^c^	74.0 ± 0.4^ac^	79.6 ± 0.5^abc^
SBP (mmHg)	102.5 ± 0.5	113.9 ± 0.8^a^	116.0 ± 0.5^ab^	101.4 ± 0.4^c^	108.7 ± 0.6^ac^	110.8 ± 0.6^abc^
DBP (mmHg)	64.1 ± 0.4	69.9 ± 0.6^a^	72.2 ± 0.4^ab^	64.3 ± 0.3	69.1 ± 0.4^a^	70.9 ± 0.4^ab^
TC (mmol/L)	4.03 ± 0.03	4.01 ± 0.04	4.11 ± 0.03	4.18 ± 0.03^c^	4.04 ± 0.04^a^	4.02 ± 0.04^a^
TG (mmol/L)*	0.79 ± 0.02	1.07 ± 0.03^a^	1.12 ± 0.02^ab^	0.96 ± 0.02^c^	1.03 ± 0.03^a^	1.18 ± 0.03^a^
HDL-C (mmol/L)	1.54 ± 0.01	1.33 ± 0.02^a^	1.27 ± 0.01^ab^	1.52 ± 0.01	1.34 ± 0.01^a^	1.27 ± 0.01^ab^
LDL-C (mmol/L)	2.40 ± 0.03	2.51 ± 0.04	2.65 ± 0.02^ab^	2.55 ± 0.03^c^	2.56 ± 0.04	2.57 ± 0.03
Glucose (mmol/L)	5.10 ± 0.02	5.16 ± 0.02	5.16 ± 0.02	4.95 ± 0.01^c^	5.05 ± 0.03^ac^	5.09 ± 0.02^ac^
Insulin (mU/L)*	6.20 ± 0.17	10.53 ± 0.44^a^	13.81 ± 0.37^ab^	7.38 ± 0.16^c^	11.73 ± 0.40^ac^	15.10 ± 0.50^abc^
Leptin (ng/mL)*	1.7 ± 0.10	7.8 ± 0.52^a^	15.3 ± 0.45^ab^	5.3 ± 0.19^c^	14.6 ± 0.61^a^	20.4 ± 0.69^abc^
Adiponectin (ug/mL)*	15.1 ± 0.35	11.4 ± 0.41^a^	10.3 ± 0.22^a^	15.0 ± 0.26	11.6 ± 0.33^a^	10.6 ± 0.26^a^
HOMA-IR*	1.43 ± 0.04	2.47 ± 0.11^a^	3.21 ± 0.09^ab^	1.65 ± 0.04^c^	2.66 ± 0.09^a^	3.45 ± 0.11^abc^

Data showing means with standard error (SE). *Skewed distributions were logarithmically transformed for comparison. Two-sample *t*-test for comparison between girls and boys in each BMI-stratified group and one-way ANOVA were used to compare characteristics across three weight groups in each sex; ^a^ *P* < 0.05 compared with normal weight (WT) group in the same sex; ^b^ *P* < 0.05 compared with overweight groups in the same sex; ^c^ *P* < 0.05 compared between boys and girls in the same weight group. SBP: systolic blood pressure, DBP: diastolic blood pressure, TG: triglyceride, TC: total cholesterol, HDL-C: high density lipoprotein cholesterol, LDL-C: low density lipoprotein cholesterol, HOMA-IR: homoeostasis model assessment of insulin resistance.

**Table 2:** Stepwise forward multiple regression analyses for Ln HOMA-IR with subjects stratified by sex and weight.

	Parameters	β ± SE	Beta	*P*
Boys (*n* = 1616)				
Normal weight (*R* ^2^ = 0.247)	Leptin*	0.227 ± 0.027	0.304	<0.001
	Tanner stage	0.178 ± 0.021	0.303	<0.001
	TG*	0.262 ± 0.066	0.149	<0.001
Overweight (*R* ^2^ = 0.274)	Leptin*	0.208 ± 0.039	0.286	<0.001
	Adiponectin*	−0.139 ± 0.061	−0.122	0.023
	Tanner stage	0.120 ± 0.033	0.224	<0.001
	TG*	0.266 ± 0.078	0.181	0.001
	SBP	0.007 ± 0.003	0.153	0.011
Obesity (*R* ^2^ = 0.288)	Leptin*	0.208 ± 0.030	0.260	<0.001
	Adiponectin*	−0.138 ± 0.040	−0.119	0.001
	Waist circumference	0.008 ± 0.003	0.127	0.006
	Tanner stage	0.067 ± 0.023	0.124	0.004
	TG*	0.215 ± 0.050	0.145	<0.001
	SBP	0.007 ± 0.002	0.143	<0.001

Girls (*n* = 1607)				
Normal weight (*R* ^2^ = 0.324)	Leptin*	0.227 ± 0.023	0.348	<0.001
	Tanner stage	0.075 ± 0.018	0.148	<0.001
	TG*	0.203 ± 0.048	0.125	<0.001
	SBP	0.009 ± 0.002	0.154	<0.001
Overweight (*R* ^2^ = 0.260)	Leptin*	0.238 ± 0.040	0.313	<0.001
	Adiponectin*	−0.145 ± 0.056	−0.133	0.010
	Tanner stage	0.070 ± 0.025	0.158	0.005
	TG*	0.224 ± 0.077	0.152	0.004
	SBP	0.010 ± 0.003	0.169	0.001
Obesity (*R* ^2^ = 0.362)	Leptin*	0.160 ± 0.037	0.198	<0.001
	Waist circumference	0.027 ± 0.003	0.408	<0.001
	TG*	0.267 ± 0.062	0.178	<0.001

Independent parameters: leptin, diponectin, Tanner stage, TG, SBP, DBP, HDL-C, waist circumference, or BMI *z*-score or FAT%; *Skewed distributions were logarithmically transformed for analysis. Abbreviations as Table 1.

**Table 3:** Stepwise forward multiple regression analyses for Ln HOMA-IR with subjects stratified by sex and pubertal development.

	Parameters	β ± SE	Beta	*P*
Boys (*n* = 1616)				
	Leptin*	0.135 ± 0.029	0.253	<0.001
Prepuberty	Waist circumference	0.013 ± 0.003	0.207	<0.001
(*n* = 704, *R* ^2^ = 0.377)	TG*	0.203 ± 0.058	0.122	<0.001
	SBP	0.010 ± 0.002	0.164	<0.001
Earlypuberty (*n* = 258, *R* ^2^ = 0.464)	Leptin*	0.226 ± 0.004	0.407	<0.001
	Waist circumference	0.014 ± 0.004	0.237	0.001
	TG*	0.229 ± 0.082	0.143	0.006
Midpuberty (*n* = 594, *R* ^2^ = 0.442)	Leptin*	0.237 ± 0.019	0.515	<0.001
	TG*	0.299 ± 0.052	0.200	<0.001
	Adiponectin*	−0.123 ± 0.038	−0.103	0.001
Late-puberty (*n* = 60, *R* ^2^ = 0.325)	Leptin*	0.137 ± 0.064	0.252	0.038
	TG*	0.650 ± 0.182	0.422	0.001

Girls (*n* = 1607)				
	Leptin*	0.124 ± 0.037	0.234	0.001
Pre-puberty	Waist circumference	0.029 ± 0.006	0.365	<0.001
(*n* = 299, *R* ^2^ = 0.483)	TG*	0.243 ± 0.077	0.146	0.002
	SBP	0.006 ± 0.003	0.107	0.022
	Leptin*	0.153 ± 0.049	0.263	0.002
Earlypuberty	Waist circumference	0.019 ± 0.007	0.235	0.006
(*n* = 206, *R* ^2^ = 0.396)	TG*	0.267 ± 0.100	0.163	0.008
	SBP	0.010 ± 0.003	0.175	0.003
	Leptin*	0.263 ± 0.029	0.389	<0.001
Mid-puberty	Waist circumference	0.017 ± 0.004	0.254	<0.001
(*n* = 789, *R* ^2^ = 0.330)	TG*	0.244 ± 0.069	0.169	<0.001
	SBP	0.006 ± 0.003	0.018	0.032
	Leptin*	0.204 ± 0.043	0.271	<0.001
Latepuberty	Waist circumference	0.017 ± 0.004	0.254	<0.001
(*n* = 313, *R* ^2^ = 0.340)	TG*	0.244 ± 0.069	0.169	<0.001
	SBP	0.006 ± 0.003	0.108	0.032

Independent parameters: leptin, diponectin, TG, SBP, DBP, HDL-C, waist circumference, or BMI *z*-score or FAT%. *Skewed distributions were logarithmically transformed for analysis. Abbreviations as Table 1.
